# Visual outcomes and safety after bilateral implantation of a trifocal presbyopia correcting intraocular lens in a Korean population: a prospective single-arm study

**DOI:** 10.1186/s12886-020-01549-z

**Published:** 2020-07-15

**Authors:** Tae-im Kim, Tae-Young Chung, Myoung Joon Kim, Kyounghwa Lee, Joon Young Hyon

**Affiliations:** 1 Department of Ophthalmology, Yonsei University College of Medicine, Seoul, South Korea; 2 Department of Ophthalmology, Samsung Medical Center, Sungkyunkwan University School of Medicine, Seoul, South Korea; 3 Renew Seoul Eye Clinic, Seoul, South Korea; 4 Alcon Korea Ltd, Seoul, South Korea; 5 Department of Ophthalmology, Seoul National University Bundang Hospital, 82 Gumi-ro 173beon-gil, Bundang-gu, Seongnam, 13620 South Korea

**Keywords:** Intraocular lens, Satisfaction, Spectacle independence, Trifocal, Visual acuity

## Abstract

**Background:**

To investigate the 3-month postoperative performance and safety after implantation of a trifocal intraocular lens (IOL) in a Korean population.

**Methods:**

This was a clinical, prospective, multicenter, single-arm study. Forty-four subjects (88 eyes) with bilateral cataract with expected postoperative corneal astigmatism of < 1.00 diopter (D) and no ocular disease or eye condition underwent bilateral implantation of the AcrySof IQ® PanOptix IOL (TFNT00). Postoperative examination at 3 months included binocular defocus curve; binocular best corrected distance visual acuity (BCDVA); monocular/binocular uncorrected VA (UCVA) at distance (4 m), intermediate (60 cm), and near (40 cm); contrast sensitivity under photopic conditions with/without glare; and subjective outcomes, including satisfaction and spectacle independence.

**Results:**

Binocular defocus curve at 3 months after bilateral implantation showed VA of 0.1 logMAR or better from + 0.5 D through − 2.5 D. Binocular BCDVA mean ± SD at 4 m was − 0.05 ± 0.07 logMAR. Binocular and monocular UCVA was 0.03 ± 0.1 and 0.08 ± 0.12 logMAR (4 m), − 0.00 ± 0.11 and 0.05 ± 0.13 logMAR (60 cm), and 0.03 ± 0.12 and 0.09 ± 0.13 logMAR (40 cm), respectively. Contrast sensitivity with glare was 1.67 ± 0.13, 1.91 ± 0.17, 1.54 ± 0.21, and 1.14 ± 0.20 log units at 3, 6, 12, and 18 cycles/degree, respectively. At near and intermediate distances, 84 and 77% of subjects reported good/excellent satisfaction, and 84 and 91% of subjects reported spectacle independence, respectively.

**Conclusions:**

In a Korean population, visual performance of the trifocal TFNT00 IOL 3 months postoperatively was < 0.1 logMAR for binocular UCVA at all distances, with high subject satisfaction and spectacle independence.

**Trial registration:**

www.ClinicalTrials.gov (NCT03268746). Registered August 31, 2017.

## Background

Many subjects who receive monofocal intraocular lenses (IOLs) ultimately require corrective glasses after cataract surgery to improve their intermediate or near distance vision [1]. Most multifocal IOLs can produce 2 foci for distance and near vision, providing a more complete range of vision compared with monofocal IOLs [2, 3]; however, glasses may be needed for intermediate vision [3, 4]. Because many daily activities, such as viewing computer or smartphone screens, are performed at intermediate distances [5, 6], trifocal IOLs with 3 focal points have been developed to address the need for improved intermediate vision after cataract surgery [7].

The first generation of trifocal IOLs, including AT LISA® tri 839MP (Carl Zeiss Meditec, Jena, Germany) and FineVision® Micro F (PhysIOL, Liège, Belgium), has an intermediate focal point at 80 cm [8, 9]. However, for many people, the optimal distance for daily intermediate vision tasks is at arm’s length, approximately 60 to 70 cm for populations of average height [6, 10]. The AcrySof® IQ PanOptix® IOL model TFNT00 (Alcon Vision LLC, Fort Worth, TX, USA) has near and distance focal points similar to a conventional multifocal IOL and an intermediate focal point at 60 cm [11, 12]. In an optical bench study, TFNT00 provided better image quality at intermediate distance compared with either AT LISA tri 839MP or FineVision Micro F because of improved light utilization [13]. Clinical studies of TFNT00 have shown that subjects achieved visual acuity (VA) of 20/25 or better from near (40 cm) through intermediate distance (60 cm) 6 to 12 months after IOL implantation [14–16]. The results of these studies indicate that the 60-cm focal point may provide optimal intermediate vision compared with the 80-cm focal point of earlier-generation trifocal IOLs.

The popularity of cataract surgery and IOL implantation has increased in Korea over the past decade, and multifocal IOLs are the most frequently selected lenses [17]. The TFNT00 IOL may provide good VA at intermediate distance for Korean subjects, for whom average arm length is between 53 and 59 cm [18, 19]; however, no clinical studies have been conducted in this population.

The purpose of this study was to evaluate the safety and effectiveness of TFNT00 in a Korean population 3 months after implantation, including visual performance, quality of vision, and subject satisfaction of postoperative vision.

## Methods

### Intraocular Lens

The TFNT00 IOL is intended for implantation in the capsular bag to correct presbyopia after cataract surgery [12]. TFNT00 is a single-piece, ultraviolet-absorbing, and blue-light-filtering IOL with a 13.0-mm overall diameter and a 6.0-mm biconvex optic. The anterior surface of the IOL has 0.1-μm negative spherical aberration to compensate for the positive spherical aberration of an average cornea. The multifocal diffractive structure in the central 4.5-mm portion of the anterior surface of the optical zone divides incoming light to create + 2.17 diopter (D) (intermediate) and + 3.25 D (near) add powers. Cataract surgery was performed following surgeons’ routine procedures. Clear corneal incisions (1.8 to 2.75 mm) were made either on temporal or on steep axis. After phacoemulsification, implantation of the TFNT00 IOL was carried out according to the local guidelines and product information provided by Alcon Vision LLC [12].

### Study design and population

This prospective, single-arm, unmasked, nonrandomized, multicenter study enrolled subjects aged > 20 years requiring bilateral cataract surgery. The study was conducted at 4 sites in Korea: Samsung Medical Center (*n* = 15), Asan Medical Center (*n* = 12), and Severance Hospital (n = 15) in Seoul and Seoul National University Bundang Hospital (*n* = 10) in Seongnam-si. Eligible subjects were those without ocular disease that could confound study outcomes who wanted an IOL that provided near, intermediate, and distance vision. Inclusion criteria were clear intraocular media other than cataract in both eyes, calculated lens power between + 16.0 D and + 24.0 D, and preoperative or expected postoperative regular corneal astigmatism of < 1.00 D. Exclusion criteria were clinically significant corneal abnormalities; previous corneal transplantation; ocular trauma; previous refractive surgery or refractive procedures throughout the study duration; history of concurrent retinal conditions; anterior chamber ≤2.5 mm not caused by swollen cataract; concurrent anterior or posterior segment inflammation; and expectation of ocular surgical treatment, large capsulotomy, or retinal laser treatment during the study (excluding neodymium-doped yttrium aluminum garnet [Nd:YAG] capsulotomy).

Study visits included a screening visit, an operative visit for each eye, and postoperative visits at week 1 and months 1 and 3. At the screening visit, the eye with worse best corrected distance VA (BCDVA) was selected as the first operative eye; if BCDVA was the same in both eyes, the right eye was selected as the first operative eye. Implantation of the IOL in the second eye occurred within 30 days of the first eye, and according to the standard visit schedule at each participating site.

### Effectiveness endpoints

The primary endpoint was the binocular defocus curve measured 3 months after implantation. Best distance correction was varied from − 5.00 to + 2.00 D in steps of 0.50 D under photopic conditions (approximately 85 cd/m^2^), and VA was recorded at each refractive correction.

Secondary endpoints were the binocular defocus curve measured 1 month after implantation, VA at 1 and 3 months after implantation, contrast sensitivity 3 months after implantation, and responses to the subject satisfaction questionnaire at the preoperative visit and 3 months after implantation. BCDVA and mean monocular and binocular uncorrected distance VA (UCDVA, 4 m), uncorrected intermediate VA (UCIVA, 60 cm), and uncorrected near VA (UCNVA, 40 cm) were measured under photopic conditions with ambient lighting lower than chart luminance using CSV-1000 charts (distance) and Early Treatment Diabetic Retinopathy Study charts (distance, intermediate, and near). Photopic best corrected binocular contrast sensitivity was measured at 3, 6, 12, and 18 cycles per degree (cpd) using CSV-1000E charts at a distance of 2.45 m, without glare and with glare (approximately 2.5 cd/m^2^).

Subjects completed a 12-item questionnaire to determine satisfaction levels and spectacle independence. Other exploratory endpoints were photopic and mesopic pupil size 3 months after implantation, measured with a pupilometer to the nearest 0.5 mm at distance, and manifest refraction spherical equivalent (MRSE) at week 1, month 1, and month 3 after implantation, measured under photopic conditions at 2.45 m in steps of 0.25 D.

### Safety analyses

Ocular nonserious and serious adverse events (AEs), including secondary surgical interventions related to the optical properties of the IOL, were assessed for ≤3 months after implantation and coded using the *Medical Dictionary for Regulatory Activities Version 21.0*. Additional safety endpoints included IOL tilt/decentration, intraocular pressure, surgical problems, and device deficiencies.

### Statistical analyses

Binocular effectiveness was evaluated for all subjects with successful bilateral IOL implantation (full analysis set), monocular effectiveness was evaluated for all eyes with successful IOL implantation (all-implanted analysis set), and safety data were collected for all subjects with attempted IOL implantation (safety set).

Subject demographics were summarized using descriptive statistics. Effectiveness endpoints were evaluated using a 2-sided 90% CI of the mean for VA data (logarithm of the minimum angle of resolution [logMAR]). BCDVA and monocular/binocular UCDVA, UCIVA, and UCNVA were also summarized as categorical variables by visit as percentage of subjects with 20/20, 20/25, 20/32, or 20/40 vision or better. Subjective symptom questions were summarized by visit per question as total number of observations and counts and percentages in each category. AEs were summarized as counts and percentages of eyes with ocular AEs for first and second operative eyes.

### Ethics

This clinical study was conducted under an approved Institutional Review Board protocol in accordance with the ethical principles of the Declaration of Helsinki, *ISO14155:2011 Clinical Investigation of Medical Devices for Human Subjects – Good Clinical Practice*, and *Standards for Medical Devices for Good Clinical Practice*. All subjects provided voluntary informed consent before initiation of any study procedures.

## Results

### Subject disposition

Of 52 enrolled subjects, 7 discontinued the study before IOL implantation because of screen failure. Most subjects (84%) were aged < 65 years and female (Table 1). Of the 45 subjects who received TFNT00, 1 subject withdrew from the study after the first eye implantation and did not receive an IOL in the second eye. The implanted eye was included in the all-implanted and safety analysis sets; the subject was excluded from the full analysis set.

**Table 1:** Demographics and Baseline Characteristics (Full Analysis Set)

Characteristic	TFNT00 (***n*** = 44)
Age, mean (SD), y	60 (8)
Sex, n (%)	
Female	33 (75)
Race, n (%)	
Asian	44 (100)
Height, mean (SD), cm	159.9 (8.3)
Arm length, mean (SD), cm	54.2 (6.3)
Axial length, mean (SD), mm	
First eye	23.6 (0.67)
Second eye	23.6 (0.67)
Corneal astigmatism, mean (SD), D	
First eye	0.62 (0.32)
Second eye	0.57 (0.25)
Monocular BCDVA, mean (SD), logMAR	
First eye	0.26 (0.29)
Second eye	0.10 (0.18)
MRSE, mean (SD), D	
First eye	0.36 (1.48)
Second eye	0.30 (1.57)

*BCDVA* Best corrected distance visual acuity, *D* Diopter, *logMAR* Logarithm of the minimum angle of resolution, *MRSE* Manifest refraction spherical equivalent

### Effectiveness

**Fig. 1:**
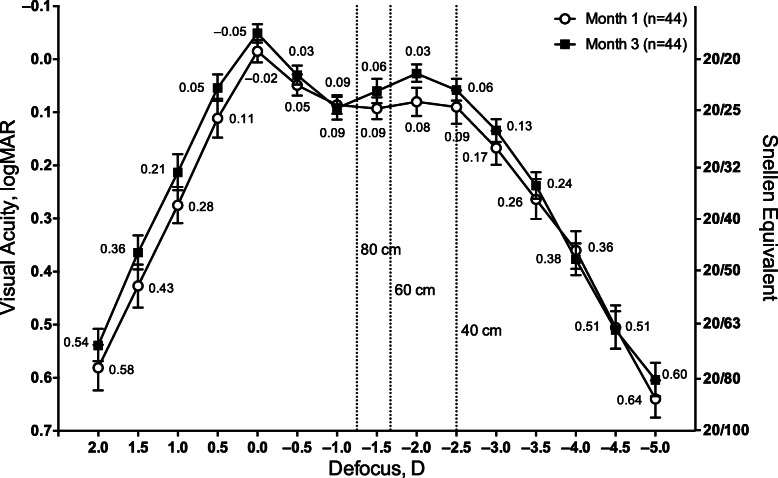
Binocular defocus curves 1 and 3 months after implantation of TFNT00. Error bars represent 90% CI. D = diopter; logMAR = logarithm of the minimum angle of resolution

At month 3 after implantation, the binocular defocus curve showed mean VA of 0.1 logMAR (20/25 Snellen) or better between + 0.50 and − 2.50 D defocus (Fig. 1). Overall, the binocular defocus curve showed that TFNT00 provided functional VA across a full range of distances, with most refractive steps showing better VA at month 3 compared with month 1.

**Fig. 2:**
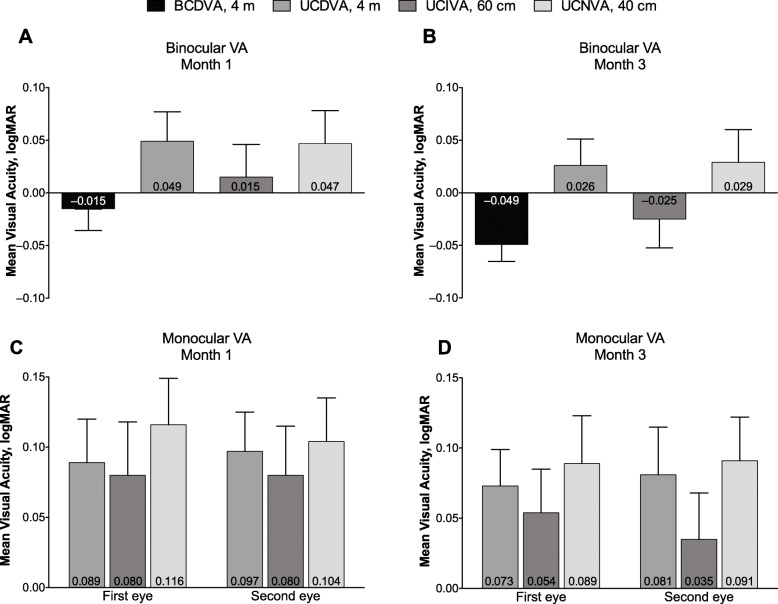
Binocular visual acuity (**a**) 1 month and (**b**) 3 months and monocular visual acuity at (**c**) 1 month and (**d**) 3 months after implantation of TFNT00 (full analysis set). Error bars represent 90% CI. BCDVA = best corrected distance visual acuity; logMAR = logarithm of the minimum angle of resolution; UCDVA = uncorrected distance VA; UCIVA = uncorrected intermediate VA; UCNVA = uncorrected near VA; VA = visual acuity

**Fig. 3:**
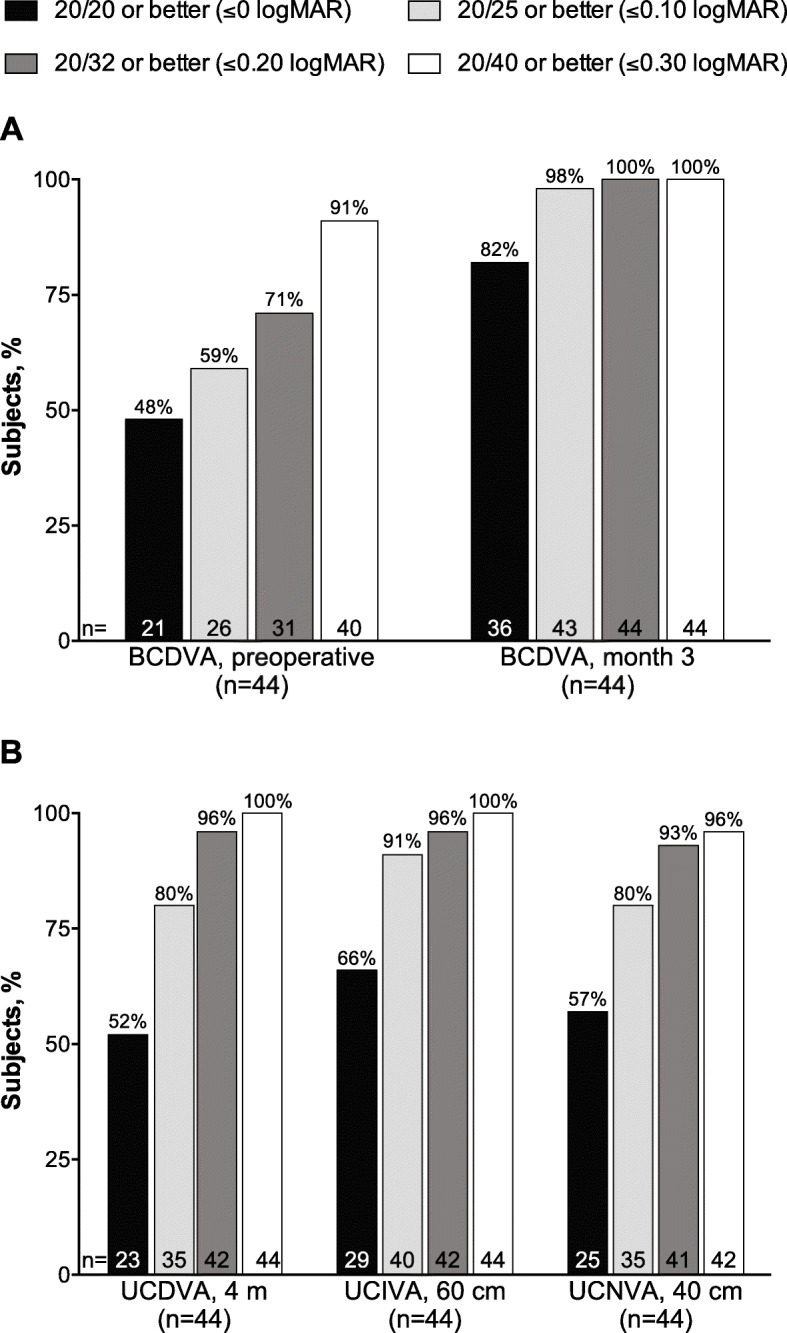
Percentages of subjects with 20/40 vision or better for (**a**) BCDVA before implantation and 3 months after implantation of TFNT00 and (**b**) UCDVA, UCIVA, and UCNVA 3 months after implantation of TFNT00 (full analysis set). BCDVA = best corrected distance visual acuity; logMAR = logarithm of the minimum angle of resolution; UCDVA = uncorrected distance VA; UCIVA = uncorrected intermediate VA; UCNVA = uncorrected near VA

**Fig. 4:**
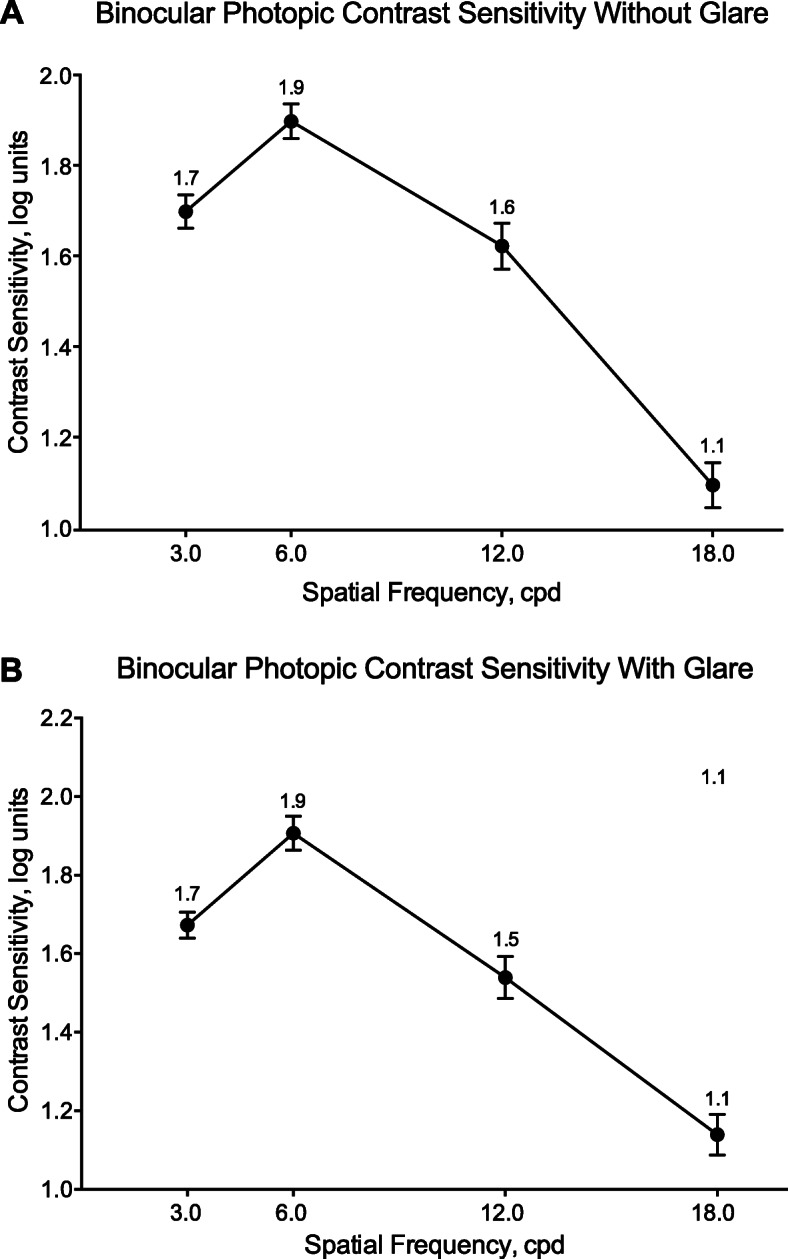
Photopic best corrected binocular contrast sensitivity at 3 months after implantation of TFNT00 (**a**) without glare and (**b**) with glare (full analysis set). Error bars represent 90% CI. cpd = cycles per degree

Binocular and monocular visual acuity are summarized in Table 2. Mean binocular BCDVA decreased from approximately 0.1 logMAR before implantation to 0.0 logMAR (20/20 Snellen) at month 1 (Fig. 2a) and month 3 (Fig. 2b) after implantation. By month 3, binocular UCVA was 0.3 logMAR or better at distance (4 m), intermediate (60 cm), and near (40 cm). Similarly, monocular UCVA improved from month 1 (Fig. 2c) to month 3 (Fig. 2d). All subjects had BCDVA 20/40 or better at month 3 compared with the preoperative visit (Fig. 3a). Most subjects had 20/40 vision or better at month 3 for binocular UCDVA (100%), UCIVA (100%), and UCNVA (96%) (Fig. 3b). Mean photopic best corrected contrast sensitivity was similar for conditions without glare (Fig. 4a) or with glare (Fig. 4b) and was highest for 6 cpd.

**Table 2:** Mean Binocular and Monocular Visual Acuity. (All-Implanted Analysis Set)

**Mean binocular visual acuity, logMAR**	**Month 1**		**Month 3**	
BCDVA, 4 m	− 0.02		−0.05	
UCDVA, 4 m	0.05		0.03	
UCIVA, 60 cm	0.02		−0.03	
UCNVA, 40 cm	0.05		0.03	
**Mean monocular visual acuity, logMAR**	**Month 1**		**Month 3**	
	**First eye**	**Second eye**	**First eye**	**Second eye**
UCDVA, 4 m	0.09	0.10	0.07	0.08
UCIVA, 60 cm	0.08	0.08	0.05	0.04
UCNVA, 40 cm	0.12	0.10	0.09	0.09

*BCDVA* Best corrected distance visual acuity, *logMAR* Logarithm of the minimum angle of resolution, *UCDVA* Uncorrected distance visual acuity, *UCIVA* Uncorrected intermediate visual acuity, *UCNVA* Uncorrected near visual acuity

Overall, after implantation of TFNT00, subject satisfaction was higher for near and intermediate vision compared with distance vision (Table 3). Before surgery, 89 and 86% of subjects were dissatisfied with their near and intermediate vision, respectively. At month 3 after IOL implantation, 84 and 77% of subjects were satisfied with their near and intermediate vision, respectively. Spectacle independence for distance, intermediate, and near vision increased by > 60% after IOL implantation. Of the 2 subjects who reported being “very dissatisfied” with surgery results at month 3, 1 experienced mild posterior capsule opacification that was not resolved and the other experienced visual impairment, conjunctivitis, corneal edema, and dry eye.

**Table 3:** Responses to Subject Satisfaction Questions (Full Analysis Set)

		TFNT00 (***n*** = 44)	
Question	Response	Preoperative ***n*** (%)	Month 3 ***n*** (%)
How satisfied are you with your vision for seeing objects at near distance?	Dissatisfied/very dissatisfied	39 (89)	3 (7)
	Neither satisfied nor dissatisfied	3 (7)	4 (9)
	Satisfied/very satisfied	2 (5)	37 (84)
How often do you wear eyeglasses or contact lenses for seeing objects at near distance?	None of the time	7 (16)	37 (84)
	Some of the time	7 (16)	6 (14)
	Most of the time	11 (25)	0
	All of the time	19 (43)	1 (2)
How satisfied are you with your vision for seeing objects at intermediate distance?	Dissatisfied/very dissatisfied	38 (86)	5 (11)
	Neither satisfied nor dissatisfied	3 (7)	5 (11)
	Satisfied/very satisfied	3 (7)	34 (77)
How often do you wear eyeglasses or contact lenses for seeing objects at intermediate distance?	None of the time	9 (21)	40 (91)
	Some of the time	12 (27)	3 (7)
	Most of the time	11 (25)	0
	All of the time	12 (27)	1 (2)
How satisfied are you with your vision for seeing objects at distance?	Dissatisfied/very dissatisfied	28 (64)	4 (9)
	Neither satisfied nor dissatisfied	11 (25)	9 (21)
	Satisfied/very satisfied	5 (11)	31 (70)
How often do you wear eyeglasses or contact lenses for seeing objects at distance?	None of the time	14 (32)	42 (96)
	Some of the time	9 (21)	1 (2)
	Most of the time	5 (11)	0
	All of the time	16 (36)	1 (2)
How often do you experience halos?	None of the time	10 (23)	3 (7)
	Some of the time	20 (46)	11 (25)
	Most of the time	10 (23)	15 (34)
	All of the time	4 (9)	15 (34)
How severe were these halos?	None	9 (21)	3 (7)
	Mild	13 (30)	5 (11)
	Moderate	15 (34)	22 (50)
	Severe	7 (16)	14 (32)
If you currently drive, how much difficulty do you have driving at night?	No difficulty at all	5 (11)	4 (9)
	A little difficulty	5 (11)	5 (11)
	Moderate difficulty	13 (30)	11 (25)
	Extreme difficulty	11 (25)	10 (23)
	I do not drive at night	10 (23)	14 (32)
If you do not drive at night, what is the reason?	Because of your current eyesight	12 (27)	11 (25)
	Because you are not interested in driving	5 (11)	5 (11)
	Because you have other reasons	10 (23)	12 (27)
	I drive at night	17 (39)	16 (36)
How satisfied are you with your cataract surgery result?	Dissatisfied/very dissatisfied	N/A	3 (7)
	Neither satisfied nor dissatisfied		8 (18)
	Satisfied/very satisfied		33 (75)
Would you recommend the cataract surgery and the new lenses that were put into your eyes to other people?	No	N/A	16 (36)
	Yes		28 (64)

*N/A* Not applicable

Although the study sample size was relatively small, a range of pupil sizes were observed in the all-implanted analysis set (Table 4). Subgroup analysis of the defocus curve by pupil size at month 3 did not show an effect of photopic pupil size on visual performance at any range of defocus.

**Table 4:** Photopic and Mesopic Pupil Size 3 Months Post-Implantation (All-Implanted Analysis Set)

	TFNT00	
	First Eye (***n*** = 45)	Second Eye (***n*** = 44)
Photopic pupil size, mean (SD), mm	3.84 (0.71)	3.82 (0.84)
Photopic pupil size category, n (%)		
Small (≤2.5 mm)	0	2 (5)
Medium (2.5–4 mm)	27 (61)	25 (57)
Large (≥4 mm)	17 (39)	17 (39)
Mesopic pupil size, mean (SD), mm	5.31 (0.95)	5.26 (1.01)
Mesopic pupil size category, n (%)		
Small (≤2.5 mm)	0	0
Medium (2.5–4 mm)	4 (9)	3 (7)
Large (≥4 mm)	40 (91)	41 (93)

**Fig. 5:**
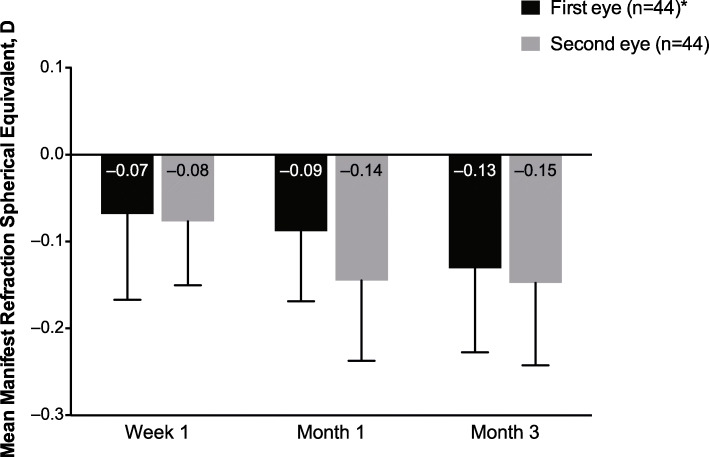
Mean manifest refraction spherical equivalent over time after implantation of TFNT00 (all-implanted analysis set). Error bars represent 90% CI. D = diopter. *At week 1, *n* = 45 for the first eye.

After IOL implantation, mean MRSE was approximately − 0.1 D throughout the study period (Fig. 5). By month 3, absolute residual refraction was within 0.3 D of the target MRSE, indicating good refractive predictability of TFNT00.

### Safety

The most common AEs were dry eye (24%) and glare (22%). All other AEs occurred in < 10% of subjects (Table 5), and no subjects discontinued the study because of an AE. Although halo vision occurred in 7% of eyes, no subjects required secondary surgical intervention because of halos. Two serious ocular AEs were reported in 1 subject who experienced mild decentration (2 mm) of the IOL due to capsular contraction and subsequently underwent secondary surgical intervention for repositioning of the IOL.

**Table 5:** Adverse Events^a^ (Safety Set)

Preferred Term	TFNT00			
	First Eye (***n*** = 45)		Second Eye (***n*** = 44)	
	***n*** (%)	E	***n*** (%)	E
Dry eye	11 (24)	11	10 (23)	10
Glare	10 (22)	10	9 (21)	9
Visual impairment	3 (7)	4	3 (7)	3
Halo vision	3 (7)	3	3 (7)	3
Foreign body sensation in eyes	3 (7)	3	2 (5)	2
Vitreous floaters	2 (4)	2	3 (7)	3
Posterior capsule opacification	1 (2)	1	2 (5)	2
Vision blurred	1 (2)	1	2 (5)	2
Conjunctivitis allergic	1 (2)	1	1 (2)	1
Corneal abrasion	1 (2)	1	1 (2)	1
Corneal edema	1 (2)	1	1 (2)	1
Meibomian gland dysfunction	1 (2)	1	1 (2)	1
Conjunctivitis	1 (2)	1	0	0
Corneal opacity	1 (2)	1	0	0
Device dislocation	0	0	1 (2)	1
Myopia	0	0	1 (2)	1
Optic disc hemorrhage	1 (2)	1	0	0
Photopsia	1 (2)	1	0	0
Surgery	0	0	1 (2)	1

*AE* Adverse event, *E* Event, *n* Number of eyes with an event. ^a^ If an eye had multiple occurrences of an AE, the eye was presented only once in the respective eye count column for the corresponding AE. Events were counted each time in the event column. AEs were coded using the *Medical Dictionary for Regulatory Activities Version 21.0*

Clinically significant subjective posterior capsule opacification was reported in 3 eyes of 2 subjects and was assessed by the investigator as mild and not related to the IOL. One eye required an Nd:YAG laser treatment of 2-mm-diameter posterior capsulotomy. Two nonserious ocular device AEs were reported by 2 subjects: 1 subject reported mild halo vision in both eyes that resolved at month 3, and 1 subject reported mild visual impairment in both eyes that resolved without sequelae.

## Discussion

Subjects who receive IOLs increasingly expect to achieve an extended range of vision after cataract surgery [20]. Compared with a standard monofocal IOL, the trifocal TFNT00 IOL had better corrected and uncorrected near and intermediate VA [21] and may be a suitable choice for subjects who want to achieve spectacle independence after cataract surgery. In 2 large multicenter clinical trials of TFNT00 with study sites located in Australia, Europe, South America, and the United Kingdom, subjects reported high levels of satisfaction with TFNT00 in addition to improved visual outcomes for near, intermediate, and distance vision [16]. Although TFNT00 has been studied in western populations, it has not been evaluated in the Korean population. Recently, the prevalence of myopia in Korea has increased [22], and ophthalmic evaluation surveys from 2008 to 2014 showed that 71% of Korean subjects aged < 50 years and 65% of children had myopia [23, 24]. In some regions, the prevalence of myopia has been reported to be > 80% [25], which may result from increased time spent performing near-distance work [23]. Consequently, many people in Korea have worn glasses since childhood, leading to high expectations for spectacle independence after cataract surgery. In addition, approximately 33% of Korean subjects undergoing cataract surgery are aged < 65 years [19], and this relatively young population wants to achieve spectacle independence after surgery for daily intermediate-distance activities such as computer work.

In this study, visual outcomes and safety were evaluated 3 months after implantation of the TFNT00 IOL in a Korean population. The intermediate focal point at 60 cm was expected to provide optimal intermediate vision for most subjects in the study, because average arm length is 54 cm. At month 3, the binocular defocus curve showed that TFNT00 provided vision of approximately 0.1 logMAR or better over a full range of defocus, and between defocus corresponding to distances of 80 to 40 cm, subjects achieved 0.06 logMAR or better. Study results showed that the Korean population had similar visual outcomes compared with those of western populations who received TFNT00. In a 12-month single-arm trial of 145 subjects in western countries, the mean ± SD best corrected intermediate VA was 0.04 ± 0.12 and 0.08 ± 0.14 logMAR at 60 cm and 80 cm, respectively, and VA of 20/25 or better was achieved across a range of distances from 4 m to 40 cm [15]. In the current study, binocular UCIVA measured at 60 cm was 0.02 logMAR 1 month after IOL implantation and improved to − 0.03 logMAR by month 3, indicating that TFNT00 provided excellent intermediate vision. Overall, 90% of Korean subjects achieved 20/25 vision or better at intermediate distance. Approximately 80% of subjects were satisfied with their postoperative vision, and spectacle independence for intermediate vision increased from 21% before IOL implantation to 91% after implantation. This finding suggests that overall subject satisfaction was improved by better intermediate vision.

In previous comparative clinical trials of TFNT00 with other trifocal IOLs, TFNT00 showed improved visual outcomes at 60 cm. A study of TFNT00 compared with the visual performance of Micro F, an earlier-generation trifocal IOL, showed better VA at 60 cm for subjects who received TFNT00 (*P* < 0.05) [14]. Furthermore, VA at preferred reading distance (approximately 42 cm) was 0.07 ± 0.07 and 0.11 ± 0.08 logMAR for TFNT00 and Micro F, respectively (*P* = 0.04) [14]. Similarly, TFNT00 showed improved performance at near distance and at preferred reading distance compared with the Tecnis Symfony (Johnson & Johnson) IOL, an extended depth-of-focus lens [26]. At 40 cm, mean VA was 0.04 ± 0.06 and 0.20 ± 0.06 logMAR for TFNT00 and Symfony, respectively (*P* < 0.001), and the VA at 60 cm for TFNT00 was 0.06 ± 0.10 logMAR [26], the results of which are comparable with those reported in the current study. The results of these studies suggest that TFNT00 may be a good choice for subjects who want to achieve spectacle independence at both intermediate and near distances.

In a large multicenter trial conducted in western countries, binocular UCIVA and UCNVA was better for subjects who received TFNT00 compared with those who received AT LISA tri 839MP (*P* = 0.002 and *P* = 0.003, respectively) 6 months after IOL implantation [16]. Recent trials of the AT LISA tri 839MP IOL, which has an intermediate focal point at 80 cm, in Korean subjects showed that AT LISA tri 839MP provided better VA at intermediate distances compared with a conventional multifocal IOL [27]; however, UCIVA was 0.13 logMAR 6 months after implantation of AT LISA tri 839MP [28]. Although future comparative studies should be conducted for Asian populations, the results of the current study indicate that visual outcomes may be improved with TFNT00 compared with AT LISA tri 839MP for subjects in Korea.

Some subjects who receive multifocal IOLs may have increased visual disturbances and reduced contrast sensitivity compared with those who receive monofocal lenses [29]. Overall, the rates of visual disturbances reported after implantation of TFNT00 in this study were consistent with those in previous studies [29]. Contrast sensitivity results showed a curve similar to that reported for healthy young subjects (mean age 21 y) with normal VA [30], indicating that TFNT00 did not cause a meaningful reduction in contrast sensitivity. Additionally, glare did not affect contrast sensitivity results, and no secondary surgical interventions were required because of visual disturbances of halo or glare.

Limitations of the present study were the relatively short follow-up period and lack of a comparison group. Future trials should evaluate the long-term outcomes, including subject satisfaction, of TFNT00 in the Korean population compared with other multifocal lens options for the correction of presbyopia. Another caveat is that in this study, we used a standard 60-cm distance to assess intermediate vision, although the average arm length in this population is 53–59 cm. Additionally, this study did not include visual quality measurements such as mesopic and scotopic contrast sensitivity, halometry, and ocular aberration; these outcomes should be evaluated in future trials.

## Conclusions

In conclusion, this study showed that Korean subjects who received TFNT00 had functional results across the full range of distance, particularly from near to intermediate, had good quality of vision at all distances, and high satisfaction. Overall, the TFNT00 IOL may provide this population with the best intermediate distance results compared with other available trifocal IOLs.

## Data Availability

The datasets used and/or analyzed during the current study are available from the corresponding author on reasonable request.

## Abbreviations

AEs: Adverse events
BCDVA: Best corrected distance visual acuity
cpd: Cycles per degree
D: Diopter
IOLs: Intraocular lenses
logMAR: Logarithm of the minimum angle of resolution
MRSE: Manifest refraction spherical equivalent
Nd:YAG: Neodymium-doped yttrium aluminum garnet
UCDVA: Uncorrected distance visual acuity
UCIVA: Uncorrected intermediate visual acuity
UCNVA: Uncorrected near visual acuity
VA: Visual acuity
